# Fungicide and warming interact to reduce soil ecosystem functioning

**DOI:** 10.1093/ismejo/wrag196

**Published:** 2026-07-26

**Authors:** Matthew Kelbrick, Jake McEntagart, James McCartney, Edward Cairns, Steve Paterson, Sharon E Zytynska, James P J Hall, Siobhan O’Brien

**Affiliations:** Department of Evolution, Ecology and Behaviour, Institute of Infection, Veterinary and Ecological Sciences, University of Liverpool, Liverpool L69 7ZB, United Kingdom; Moyne Institute of Preventive Medicine, Department of Microbiology, School of Genetics and Microbiology, Trinity College Dublin, Dublin 2, D02 PN40, Ireland; Moyne Institute of Preventive Medicine, Department of Microbiology, School of Genetics and Microbiology, Trinity College Dublin, Dublin 2, D02 PN40, Ireland; Department of Evolution, Ecology and Behaviour, Institute of Infection, Veterinary and Ecological Sciences, University of Liverpool, Liverpool L69 7ZB, United Kingdom; Department of Evolution, Ecology and Behaviour, Institute of Infection, Veterinary and Ecological Sciences, University of Liverpool, Liverpool L69 7ZB, United Kingdom; Department of Earth and Environmental Sciences, Faculty of Science and Engineering, The University of Manchester, M13 9PL, United Kingdom; Department of Evolution, Ecology and Behaviour, Institute of Infection, Veterinary and Ecological Sciences, University of Liverpool, Liverpool L69 7ZB, United Kingdom; Moyne Institute of Preventive Medicine, Department of Microbiology, School of Genetics and Microbiology, Trinity College Dublin, Dublin 2, D02 PN40, Ireland

**Keywords:** soil microbial communities, fungicide, pesticide, warming, climate change, adaptation, respiration, community functioning

## Abstract

Soil microbial communities are essential to the functioning of their ecosystems, providing vital services such as plant growth promotion and bioremediation. However, intensively managed environments such as agricultural soils are increasingly challenged with multiple harsh environmental stressors from the compounding effects of climate change and conventional agricultural practices. Understanding how soil communities will respond to environmental stress along multiple axes is key for predicting how environmental change will impact terrestrial soil ecosystems. We combine experimental evolution with community phenotyping, 16S rRNA gene sequencing, fungicide resistance assays, and plant experiments, to show that fungicide and warming temperatures together drive a synergistic reduction in soil community respiration rates, metabolic capacity and *Hordeum vulgare* plant shoot biomass, which would have been missed by looking at stressor effects independently. Combined warming and fungicide stress caused widespread loss of metabolic activity, particularly for carbohydrates and carboxylic acids, highlighting impairment of community function. Fungicide-only treated soil isolates had a 16-fold increase in fungicide resistance, but only in the absence of warming—suggesting that warming constrained fungicide adaptation. Together, our findings show that the number of stressors (rather than the nuances of individual stressors) may be key for predicting soil microbial community responses to environmental change across multiple axes.

## Graphical Abstract

**Graphical Abstract:**
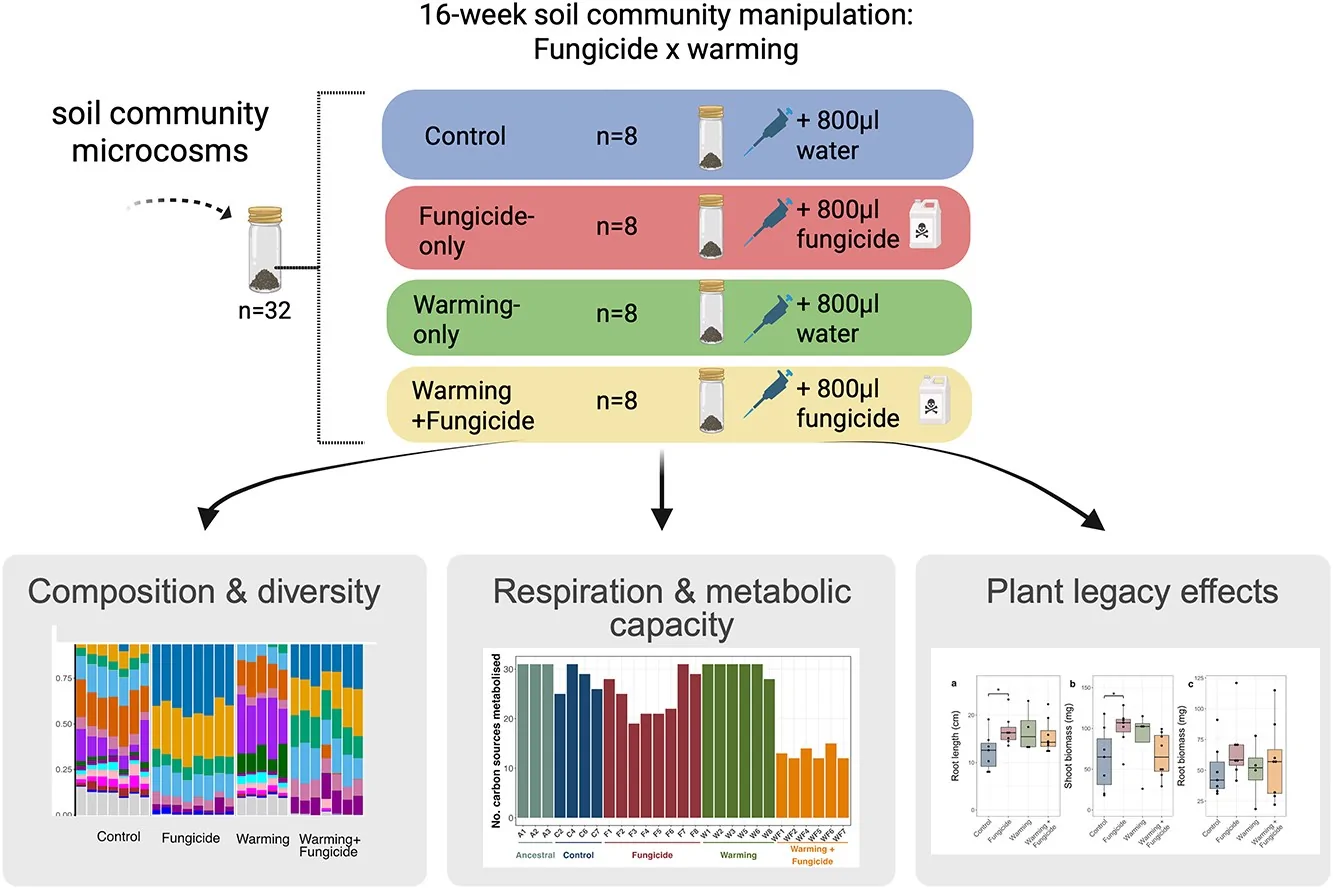

## Introduction

Soils harbour diverse and complex microbial communities that perform essential ecological functions, including nutrient cycling, carbon storage, and crop protection [1]. These activities are increasingly threatened by anthropogenic activities such as pesticide application, chemical pollution, and land-use change, which can alter soil community composition, reduce microbial diversity, and impair ecosystem functioning [2–4]. In the real world, soil environments are frequently subjected to multiple, simultaneous disturbances [2, 5]. For example, climate change-induced heatwaves may co-occur with agricultural practices such as pesticide application. Mounting evidence suggests that the combined effects of multiple stressors can yield non-additive, often unpredictable outcomes that differ from single-stressor scenarios [2, 4–8]. Nevertheless, the vast majority of studies experimentally addressing the impact of global change on microbial communities tend to examine global change factors individually [5]. Understanding how multiple stressors act on soil communities could provide information on which stressors to prioritize for maintaining ecosystem stability under climate change [7].

The growing awareness of anthropogenic pressures on terrestrial ecosystems means there is increasing demand for experimental studies investigating the interactive effects of multiple stressors on soil microbial communities, and the consequences for microbial ecosystem functioning. When multiple stressors act on a community, their combined effects can be synergistic (combined effects are more than expected), antagonistic (combine effects are less than expected), or additive (combined effects are equal to the sum of individual effects; the null model) [7]. For instance, species that tolerate one stressor may differ from those that can withstand another, and few may be capable of tolerating both. Consequently, when stressors are combined, the community may lack taxa able to persist under the joint conditions [8], so that even additive effects on species composition can have synergistic effects on functioning. Over longer timescales, evolutionary trade-offs and antagonistic pleiotropy could also underlie synergistic stressor effects [7], if adaptation to one stressor increases susceptibility to a second stressor (e.g. collateral sensitivity [9]). In contrast, cross-resistance, co-resistance, or co-regulation could underlie antagonistic effects of multiple stressors, if adaptation to one stressor reduces susceptibility to a second [10]. Multiple stressors can also reduce the evolutionary potential of populations by lowering population sizes and inducing genetic bottlenecks, which limit genetic variation and the supply of beneficial mutations [11].

Pesticides are widely used in agriculture to maintain sufficient food production amid global population growth. In 2021, 355 175 tonnes of pesticides were sold in the European Union [12]. However, only a fraction reaches target pests; estimates suggest that 30%–50% of applied pesticide is lost by deposition on the ground, or volatized [13, 14]. Pesticides may exert unintended effects on non-target organisms by targeting cell membrane components, protein synthesis, signal transduction, respiration, cell mitosis, and nucleic acid synthesis [15, 16]. Pesticides narrow microbial metabolic breadth, select for pesticide degraders and resistant strains, and accelerate nutrient loss from soils, especially when multiple classes of pesticides are applied simultaneously [3]. Studies investigating the impact of pesticides on soil microbial communities typically focus on pesticides in isolation [3, 17, 18]. However, the long-term Nash’s Field grassland experiment at Silwood Park, UK, demonstrated that interactions between pesticides and other stressors (e.g. pH) are essential for predicting shifts in soil bacterial community composition [6].

Fungicides are the most widely sold class of pesticides in the European Union [12], and their extensive use has led to residue accumulation in agricultural soils [17]. At the same time, global temperature projections indicate significant warming of soils (1°C to 6°C increase by 2100) [19]. Soil warming can significantly alter soil bacterial community diversity, composition, and functioning, favouring fast-growing heat tolerant taxa [20–22]. Moreover, warming can increase pesticide toxicity, as shown for fish, earthworms, and mosquitos [23–25]. However, it remains unclear whether simultaneous warming and pesticide exposure may interact to shape non-target soil community composition, diversity, and ecosystem functioning, as well as whether these changes matter for plants. We investigate how fungicide exposure and warming temperatures, applied as single stressors or in combination, affects diversity, composition, and functioning of soil bacterial communities. To address this question, we conducted a fully factorial soil microcosm experiment where we manipulated exposure to fungicide (Fubol Gold: Syngenta; 64% w/w mancozeb +3.88% w/w metalaxyl-M) and/or warming temperatures for 16 weeks. We assessed how the two stressors interact to shape soil community composition, diversity, functioning (CO_2_ production and metabolic capacity across 31 carbon sources), adaptation (fungicide resistance), vegetative stage plant growth promotion (Spring Barley, *Hordeum vulgare*, cv. Curtis), and plant herbivore population growth (*Sitobion avenae)*.

## Materials and methods

### Sixteen-week soil community experiment

*a) Extracting a soil community*

A soil microbial community was extracted from John Innes No.2 compost (J. Arthur Bowers) by hand-shaking 200 g compost with 400 sterile glass beads in 200 ml M9 buffer in a sterile 1 L Duran for 2 min. After settling for 15 min, the supernatant was collected and cryopreserved in 20% (v/v) glycerol at −80°C*.* This extracted community was used to initiate our 16-week experiment (below) and is hereafter referred to as the “ancestral community”.

*b) Establishing soil community microcosms*

Our 16-week experiment followed a 2 × 2 factorial design with fungicide (present/absent) and warming (present/absent) as fixed factors. This generated four treatment groups: Control (no fungicide, no warming), Fungicide-only (fungicide, no warming), Warming-only (warming, no fungicide), and Warming+Fungicide (warming and fungicide), each replicated eight times (*n* = 8), giving 32 soil microcosms in total (Fig. S1).

Sterile soil microcosms were first prepared by twice-autoclaving (121°C for 20 min, twice) 30 ml glass universal tubes containing 10 g compost. After autoclaving, sterile soil microcosms were left at room temperature for 5 days to allow reoxidation of sterilization by-products [26], before adding 100 μl of thawed ancestral community supernatant (Supplementary Methods SM1.1). We chose to sterilize and re-add the soil community (rather than simply begin with unsterilized soil) because later functional tests require soil sterilization to generate common garden conditions (Sections 2.3, 2.4 and 2.5).

Fubol Gold fungicide was added to 16 microcosms allocated to our two fungicide treatments (eight microcosms for the Fungicide-only treatment, and eight microcosms for the Warming+Fungicide treatment). Fungicide was first dissolved in sterile water to a concentration of 1 g ml^−1^, before diluting to the relevant working concentration (10 mg kg^−1^ at Week 1). Fungicide concentrations increased at 4-week intervals during the 16-week experiment to simulate fungicide accumulation in soil (Weeks 1–4: 10 mg kg^−1^, Weeks 5–8: 50 mg kg^−1^, Weeks 9–12: 100 mg kg^−1^, Weeks 13–16: 500 mg kg^−1^). Microcosms were treated with 800 μL fungicide solution, vortexed for 30 s and incubated statically (26°C, 70% relative humidity (RH)). The remaining 16 fungicide-free microcosms (eight microcosms for the Control treatment, and eight microcosms for the Warming-only treatment), were treated with 800 μL sterile water. As our experiments were conducted as closed systems, it was not our principal aim to directly mimic natural concentrations of fungicide found in agricultural soils (because these can vary widely across locales and season), but to simulate strong directional selection for fungicide resistance throughout the experiment, analogous to what microbial communities could experience in the real world. Control and Fungicide-only microcosms were maintained at 26°C throughout the experiment, whereas Warming-only and Warming+Fungicide microcosms were subjected to a weekly temperature increase of 0.5°C to a maximum of 34°C (Fig. S1).

Every 7 days, for 16 weeks, all 32 microcosms were washed with 10 ml sterile M9, vortexed with 20 sterile 5 mm glass beads, before transferring 100 μL of soil supernatant to fresh sterile soil. This “transfer protocol” was designed to simulate mixing and replenishment of resources such as would occur naturally in a soil patch exposed to weather, plants, and animals, and is a well-established approach for experimental evolution [27, 28]. Week-16 soil supernatants were plated on Luria-Bertani (LB) agar to estimate abundance, and supernatants were cryopreserved at −80°C with, or without, 20% (w/v) glycerol. These 32 cryopreserved week-16 communities are hereafter referred to as “week-16 communities”.

Week-16 community supernatants cryopreserved without glycerol were used for 16 s rRNA gene sequencing [29, 30] (Section 2.2), and week-16 community supernatants cryopreserved with glycerol were used for CO_2_ production (Section 2.3), metabolic profiling (Section 2.4), plant growth assays (Section 2.5), and fungicide minimal inhibitory concentration (MIC) assays (Section 2.6). Week-16 communities were each allocated a unique code: a single letter C, F, W, or WF (corresponding to Control, Fungicide-only, Warming-only, or Warming+Fungicide, respectively), followed by replicate ID (1–8). For example, replicate five from fungicide-only communities was given the ID “F5”. Soil supernatants had been cryopreserved for ~1 year prior to functioning assays and rRNA gene sequencing.

### Community 16S ribosomal RNA (rRNA) gene sequencing

To analyse taxa and diversity shifts in week-16 bacterial communities, we performed DNA extraction and 16S rRNA gene sequencing. All 32 week-16 soil supernatants (eight replicates from each of four treatments: Control, Fungicide-only, Warming-only, and Warming+Fungicide) were thawed, and DNA extracted using a Qiagen DNeasy PowerSoil Pro Kit. DNA quantity and quality were measured using a nanodrop. Sequencing was performed by the Centre for Genomic Research (Liverpool, UK) using a MiSeq System (Illumina, 2 × 150 bp). Bacterial 16S rRNA gene libraries were prepared using the NimaGen EasySeq RC-PCR 16S rRNA Bacterial ID Kit. Multiple primer pairs were used targeting the V1–V2, V3, V4, V5, V6, and V9 regions of the bacterial 16S rRNA gene [31].

Reads were demultiplexed, adapter-trimmed (Cutadapt v1.2.1), quality-checked (FastQC v0.12), and trimmed with Sickle (v1.200; Q ≥ 20, min length 15 bp). Sequences were denoised with DADA2 [32] within QIIME 2 v2023.2 [33], trimmed to 150 bp, and chimeras removed. Taxonomy was assigned using the SILVA classifier v138.1 [34], and phylogenetic analyses were conducted with MAFFT and FastTree within QIIME 2 [35]. Three samples (C5, W8, and WF8) fell below the rarefaction cutoff (<120 000 reads) and were therefore excluded from the 16S rRNA gene dataset. We did not detect any evidence of a community in two samples (W4 and W7), with the exception of one *Pseudomonas* taxon, suggesting that only our *Pseudomonas fluorescens* strain, and not the ancestral community, was added to these two microcosms at the beginning of the 16-week experiment (Supplementary methods SM1.1). Communities W4 and W7 were therefore also excluded from the 16S rRNA gene dataset and all subsequent experiments.

### Community functional profiling: CO_2_ production

Respiration (CO_2_ production), used here as a proxy for soil metabolic activity [36] was quantified for all 32 week-16 soil supernatants (eight replicates from each of four treatments: Control, Fungicide-only, Warming-only, and Warming+Fungicide), using MicroResp [35]. To test how the background soil experimental conditions altered CO_2_ production more generally, we also included the ancestral soil community supernatant in our assay. Fresh compost was sieved (~1.2 mm) and 0.35 g was added to all wells of a deep-well 96-well plate, covered with a gas-permeable metal lid, twice-autoclaved (126°C, 15 min), and rested for 5 days. 50 μL sterile water was added to each well, vortexed for 30 s and left for 1 h before community re-inoculation. Respiration assays and community metabolic profiling (see Section 2.4) were run concurrently. Cryopreserved soil communities were revived by inoculating 100 μL thawed soil supernatant into 10 g sterile soil in 30 ml glass universals and incubated under control conditions (no fungicide, 26°C) for 14 days, with transfer to fresh, sterile soil after 7 days (washed with 10 ml sterile M9, vortexed with 20 sterile 5 mm glass beads, before transferring 100 μL of soil supernatant to 10 g fresh sterile soil). At Day 14, 10 ml sterile M9 was added to each glass universal, vortexed with 20 glass beads, and allowed to settle for 1 h. The supernatant (50 μL) was then transferred into 0.35 g sterilized soil in a deep-well 96-well plate, prepared previously. The 96-well plate was vortexed, and incubated at 26. After 7 days, respiration was measured over 24 h at 26°C using a colorimetric detection lid (Supplementary methods SM1.2), with optical density (OD) at 580 nm recorded at 0 h and 24 h. The respiration detection lid contained a colorimetric dye, which responds to pH change caused by CO_2_ production by changing from pink to yellow. Higher OD values indicated lower respiration, as the dye remained pink. ΔOD_580_ was calculated for each community by subtracting OD_580_ at 24 h from OD_580_ at 0 h_,_ with higher values indicating greater respiration. Respiration assays were performed in duplicate for each week-16 community and in triplicate for the ancestral community. Microbial population densities were not standardized between community samples prior to the respiration assay to avoid disproportionately removing rare taxa, which could artificially influence ecosystem functioning [37, 38].

### Community functional profiling: metabolic capacity

Metabolic profiles were assessed using Biolog EcoPlates (Biolog Inc., USA), which contain 31 different carbon substrates and a water control (Table S1). We analysed substrate utilization across 31 carbon sources for all 32 week-16 communities (eight replicates from each of four treatments: Control, Fungicide-only, Warming-only and Warming+Fungicide), and the ancestral community. Cryopreserved soil communities were revived by inoculating 100 μL thawed soil supernatant into 10 g sterile soil in 30 ml glass universals and incubating under control conditions (no fungicide, 26°C) for 14 days, with passage to fresh sterile soil after 7 days. At Day 14, 10 ml M9 buffer was added to each soil microcosm, vortexed with glass beads and 1 ml supernatant was centrifuged (1 min, 500 g). Supernatants were diluted 10× in sterile 0.85% NaCl, and aliquots of 150 μL diluted supernatant were inoculated into each of 32 wells of a Biolog plate (31 carbon sources plus a water control). Plates were incubated at 26°C for 8 days. ΔOD_580_ was measured on Day 8 using a Tecan Nano plate reader. Community densities were not standardized prior to inoculation, because diluting would remove rare taxa and artificially alter community structure. Week-16 communities were assayed once; ancestral and uninoculated controls were run in triplicate.

### Plant performance assays

We tested how week-16 communities (eight replicates from each of four treatments: Control, Fungicide-only, Warming-only, and Warming+Fungicide) influenced (i) barley (*H. vulgare*, cv. Curtis) growth during the vegetative stage and herbivore population growth rates of a sap-feeding agricultural aphid pest (*S. avenae*) [39]. Growth during the initial vegetative stage is correlated with later plant health [39]. Cryopreserved week-16 soil communities were revived by inoculating 100 μL thawed supernatant into 10 g sterilized soil in 30 ml glass universal tubes and incubating for 14 days. After 14 days, each 10 g soil portion was transferred to an empty individual 20 oz plant pot with a mesh lid. As before, community densities were not standardized prior to inoculation to avoid losing rare species in higher-density communities. Surface-sterilized Curtis seeds were planted 1 cm deep, watered (3 ml) and grown at 26°C, 60% RH, 16:8 light–dark. On Day 6, two fourth-instar aphids were added to each plant. Before adding to the plant, aphids were pre-acclimated for 7 days in a growth chamber set to 26°C. The growth-promotion capability of each week-16 soil community was assayed in triplicate (32 communities ×3 replicates = 96 plants).

Plant height and adult aphid numbers were measured on Days 16, and 20. On Day 20, roots and shoots were harvested, dried at 50°C for 5 days, and weighed to determine dry biomass. Due to space constraints, we could not include the ancestral community in this experiment. Instead, Control communities acted as a baseline from which the effect of Fungicide, Warming and the interaction could be tested.

### Quantifying fungicide resistance for community isolates

To quantify fungicide resistance, we performed fungicide minimum inhibitory concentration (MIC) assays on individual isolates recovered from week-16 soil communities. MIC assays were conducted on isolates rather than whole communities because community-level measurements would primarily reflect the growth of the most resistant organism. To keep the experiment tractable, we selected three week-16 communities at random from each experimental treatment (Control, Fungicide-only, Warming-only, and Warming+Fungicide), yielding 12 communities in total. From each community, six culturable isolates were recovered (18 isolates per treatment; 72 isolates in total; Supplementary Methods SM1.3). Isolates were grown from frozen stocks overnight in LB broth, standardized to ~1.5 × 10^8^ Colony Forming Units (CFU) ml^−1^, and 10 μL added to each well of a 96-well plate containing Mueller–Hinton (MH) broth and ×2 serial dilutions of Fubol Gold fungicide (decreasing concentrations from 5 mg ml^−1^ to 0.00975 mg ml^−1^ in two-fold dilutions). Plates were incubated statically at 28°C for 18 h. Three technical replicates were performed for each isolate. The MIC was taken as the minimum concentration (mg ml^−1^) of fungicide which resulted in no detectible bacterial growth.

### Statistical analysis

All statistical analyses were carried out in R 4.5.1 [40], unless stated otherwise.

*16S rRNA gene sequencing*: Weighted and unweighted UniFrac distance matrices were generated in QIIME 2 using the QIIME “diversity core-metrics-phylogenetic” pipeline. Treatment-specific differences (Control, Fungicide-only, Warming-only, and Warming+Fungicide communities) in β-diversity were assessed using permutational multivariate analysis of variance (PERMANOVA) within QIIME 2 (beta-group-significance) on unweighted UniFrac distance matrices. Pairwise PERMANOVA tests were used for *post hoc* comparisons between treatment groups. Differentially abundant bacterial families were identified using ANCOM in QIIME 2 (*composition ancom*), with *W* values representing the number of pairwise log-ratio tests rejecting the null hypothesis of equal abundance. Principal coordinates analysis (PCoA) was performed in QIIME 2 using unweighted UniFrac distances, and ordination coordinates visualized using ggplot2 in R.

α-diversity metrics (Faith’s phylogenetic diversity and Pielou’s evenness) were generated in QIIME 2 using the “core-metrics-phylogenetic” pipeline. Statistical comparisons of alpha diversity were performed in R, using linear models (*lm*), with fungicide, warming, and their interaction included as fixed factors (diversity ~ fungicide * warming). Post hoc comparisons between treatments were conducted using pairwise *t-*tests with Benjamini–Hochberg correction, using *stat.test* () in rstatix package [41].

*Community CO_2_ production and metabolic capacity:* CO_2_ production assays and Biolog EcoPlate assays were established from the same inoculum. Six communities (C1, C3, C5, C8, WF3, WF8) failed to colonize fresh soil and showed no detectable respiration, metabolic activity, or growth on LB agar and were excluded from analyses. Two additional communities (W4, W7) were excluded because no community was detected in our 16S data (see Section 2.2).

Community CO_2_ production was analysed using generalized least squares (GLS) models using *gls* () within the nlme package [42] in R. GLS was used because residual diagnostics showed heterogeneity of variance among treatment groups. CO_2_ production (ΔOD_580_) was fitted as the response variable, with warming, fungicide and their interaction included as fixed effects. Because variation in bacterial abundance may influence community-level CO_2_ production, log_10_ (CFU g^−1^) was also included as a covariate to account for broad differences in culturable bacterial density among communities. However, we acknowledge that CFU estimates provide only a coarse proxy for total community abundance. Model terms were evaluated by comparing nested models fitted using maximum likelihood, with significance assessed using likelihood-ratio tests (LRT). We tested for differences in CO_2_ production between treatments (Control, Fungicide-only, Warming-only, Warming+Fungicide), using Dunn Kruskal–Wallis multiple comparison with Benjamini–Hochberg correction. Finally, we tested whether CO_2_ production for each treatment differed from the ancestral community using a separate Wilcoxon rank sum test for each treatment, with Benjamini–Hochberg correction.

Utilization of each carbon source in the Biolog EcoPlate was quantified as ΔOD_580_ (final − blank), with higher ΔOD_580_ values indicating greater metabolic activity on a particular carbon source. We quantified mean global metabolic potential across the 31 Biolog substrates for each community (average-well-colour-development; AWCD), calculated as the mean of all 31 ΔOD_580_ values. A higher AWCD value indicates a high mean value for substrate utilization across all 31 sources. Treatment effects on AWCD were tested using a linear model (*lm)* in R, where AWCD was fitted as the response variable, warming, fungicide and their interaction included as fixed effects, and community abundance (log_10_ (CFU g^−1^)) fitted as a covariate. Significance was assessed using F-tests of nested models. Pairwise treatment differences were analysed using analysis of variance (ANOVA) with Tukey HSD. We tested whether AWCD for each treatment differed from the ancestral community, using four separate t-tests, with Benjamini–Hochberg correction. Substrate richness was defined as the number of positive wells (OD580 > 0.25), out of 31 maximum. Community metabolic profiles were compared across Ancestral, Control, Fungicide-only, Warming-only, and Warming+Fungicide communities using PERMANOVA (adonis2, vegan [43]) based on Euclidean distances and 999 permutations. Pairwise PERMANOVA comparisons were performed using adonis2 with *P*-values adjusted with Benjamini–Hochberg correction. Ordination of community profiles was visualized using Principal Component Analysis (PCA) via *prcomp* (). Finally, metabolic activity on each substrate was compared across ancestral, Control, Fungicide-only, Warming-only, and Warming+Fungicide communities using one-way ANOVA with Benjamini–Hochberg correction, followed by t-tests with Benjamini–Hochberg correction, using *t_test* () within the rstatix package.

*Plant and aphid performance*: The germination rate of our Spring barley seeds was ~50% (47 out of 96 seeds did not germinate), which may be reflective of our experimental growth conditions which were optimized for the bacterial soil community rather than the plant (26°C, rather than 15°C–20°C) preferred by Spring barley [44]. We confirmed that germination success was not treatment-specific, using a binomial generalized linear model (GLM) with fungicide, warming, and their interaction as explanatory factors and germination as a binary response variable (1 = germinated, 0 = not germinated). Model terms were tested using likelihood ratio tests. GLM model assumptions were checked using DHARMa [45]. Because of this low germination rate, the following soil communities were excluded from the analysis due to complete failure of all three technical replicates to germinate: C7, F1, W1, and W6.

Two plants (ID 29 and 62) were identified as outliers for root biomass (> 1.5× interquartile range above the third quartile) with a root biomass value of 610 mg and 570 mg, respectively (all remaining values fell between 12 mg and 121 mg) (Fig. S2). Plant growth responses (root length, shoot biomass, and root biomass) were analysed using a multivariate analysis of variance (MANOVA) with Pillai’s trace to test effects of fungicide, warming, and their interaction. Replicate means of each soil community were for the MANOVA because technical replication was uneven (1–3 replicates per community), resulting in unbalanced sample sizes across treatments. MANOVA assumptions were assessed using Q–Q plots, and homogeneity of covariance matrices tested using Box’s M test within the biotools package in R [46, 47]. Multicollinearity among response variables was evaluated using generalized variance inflation factors (GVIF). Plant height showed high collinearity (GVIF >5) and was excluded, leaving root length, shoot biomass, and root biomass in the final model. Significant MANOVA fungicide × warming effect was followed by univariate ANOVAs and estimated marginal means using emmeans [48] for between-treatment effects.

Aphid abundance (Days 16 and 20) was analysed using negative binomial generalized linear models (glmmTMB [49]) including fungicide, temperature, day, and their interactions, with plant height included as a covariate. Community ID was fitted as a random effect to account for repeated sampling on the same aphid population. Overdispersion and residual spread was checked using DHARMa. Model simplification was conducted using likelihood ratio tests via *drop1* (). *Post hoc* comparisons of the fungicide x day interaction were performed using emmeans.

*Fungicide resistance:* Log_2_-transformed MIC values were analysed using linear mixed-effects regression (LMER) fitted with *lmer* () within the lme4 package [50], with fungicide, warming, and their interaction as fixed factors and community ID as a random effect. Model significance was assessed using likelihood ratio tests and model assumptions were checked using DHARMa.

## Results

### Fungicide alters bacterial community composition and reduces diversity

**Figure 1:**
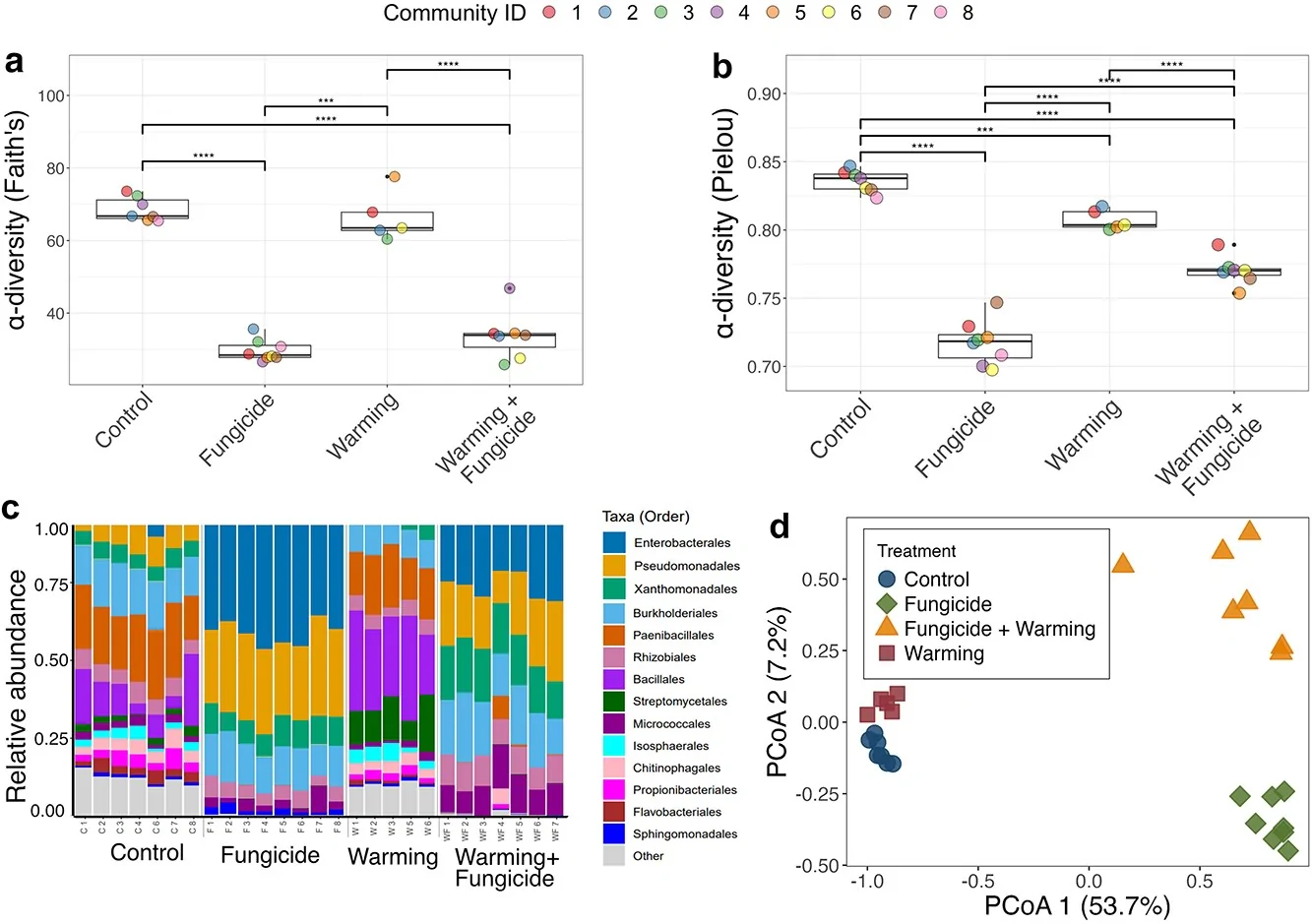
(a–d) Effects of warming and fungicide treatment on week-16 bacterial community diversity and composition assessed by 16S rRNA gene sequencing. (a) Faith’s phylogenetic diversity and (b) Pielou’s evenness, for week-16 communities grown under Control, Fungicide-only, Warming-only or Warming+Fungicide conditions. Individual points represent individual communities and are colour-coded by community ID. The median (central line), interquartile range (box), and 1.5 × interquartile range (whiskers) are shown. Lines connecting groups indicate significant pairwise differences. Significance levels are denoted as: *P.adj < .05, **P < .01, ***P < .001, ****P < .0001. (b) Relative abundance (proportion) of bacterial taxa in each week-16 community grown under Control, Fungicide-only, Warming-only or Warming+Fungicide conditions. X-axis labels show a single letter C, F, W, or WF (corresponding to Control, Fungicide-only, Warming-only or Warming+Fungicide, respectively), followed by community ID (1–8). (c) PCoA of bacterial community composition based on unweighted UniFrac distances. Percent variation explained by each axis is shown in parentheses.

Fungicide reduced bacterial α-diversity (Faith’s phylogenetic diversity; *F*_1,24_ = 309.1, *P <* .001; Fig. 1a). There was no significant fungicide x warming interaction, suggesting that the effect of fungicide on α-diversity did not depend on warming (*F*_1,23_ = 2.6, *P* > .05), and warming had no main effect on α-diversity compared to controls (*F*_1,24_ = 0.4, *P* > .05). Pairwise comparisons confirmed that Fungicide-only communities, and Warming+Fungicide communities had decreased α-diversity relative to controls (t-tests, *P*. adj < .001). However, no difference was observed in α-diversity between Fungicide-only and Warming+Fungicide communities (pairwise t-tests, *P*. adj > .05), showing that warming did not impact fungicide-driven losses in α-diversity.

Fungicide also reduced community evenness (Pielou’s index; *F*_1,24_ = 79.3, *P* < .001; Fig. 1b). Warming had no main effect on evenness (*F*_1,24_ = 3.4, *P* > .05), but there was a significant fungicide x warming interaction (*F*_1,23_ = 79.7, *P* < .001), so that warming partially mitigated the fungicide-induced decline in evenness. Fungicide-only, Warming-only, and Warming+Fungicide treated communities had lower evenness compared to controls (pairwise t-tests, *P*. adj < .001, Fig. 1b). Fungicide-only communities had lower evenness compared to Warming+Fungicide communities (pairwise t-tests, *P*. adj < .001), further supporting the finding that warming partially alleviated the negative impact of fungicide on evenness. However, Pielou’s evenness does not take phylogenetic diversity into account, treating all taxa as equally distinct.

We assessed how treatments shaped bacterial community composition using β-diversity (UniFrac distances). Treatment (Control, Fungicide-only, Warming-only, or Warming+Fungicide) significantly affected composition in both unweighted (*F* = 13.5, *P* = .001, Fig. 1c) and weighted analyses (*F* = 38.7, *P* = .001, Fig. 1c). The composition of Fungicide-only, Warming-only, and Warming+Fungicide communities all differed significantly from controls (pairwise PERMANOVA: Controls versus Warming-only *F* = 2.8, Controls versus Fungicide-only *F* = 25.5, and Controls versus Warming+Fungicide *F* = 16.4; all *P* < .001), and Warming-only and Fungicide-only communities both differed from Warming+Fungicide communities (Warming+Fungicide versus Warming-only *F* = 12.4, Warming+Fungicide versus Fungicide-only *F* = 4.4; *P* < .01 in both cases). PCoA showed treatment-specific grouping, with axis 1 (53.7% variation) separating fungicide-treated from non-fungicide treated communities (Fig. 1d), highlighting the dominant role fungicide had in shaping microbial community composition. Across 120 000 reads per sample assigned to ≤112 bacterial families, 40 (~36%) differed significantly among treatments (ANCOM; Table S2). Fungicide was associated with increased relative abundances of *Pseudomonadaceae* and *Enterobacteriaceae,* whereas warming increased relative abundance of *Streptomycetaceae* and *Bacillaceae* compared to controls (Fig. 1c, Table S2).

### Treatment-specific differences in CO_2_ production

**Figure 2:**
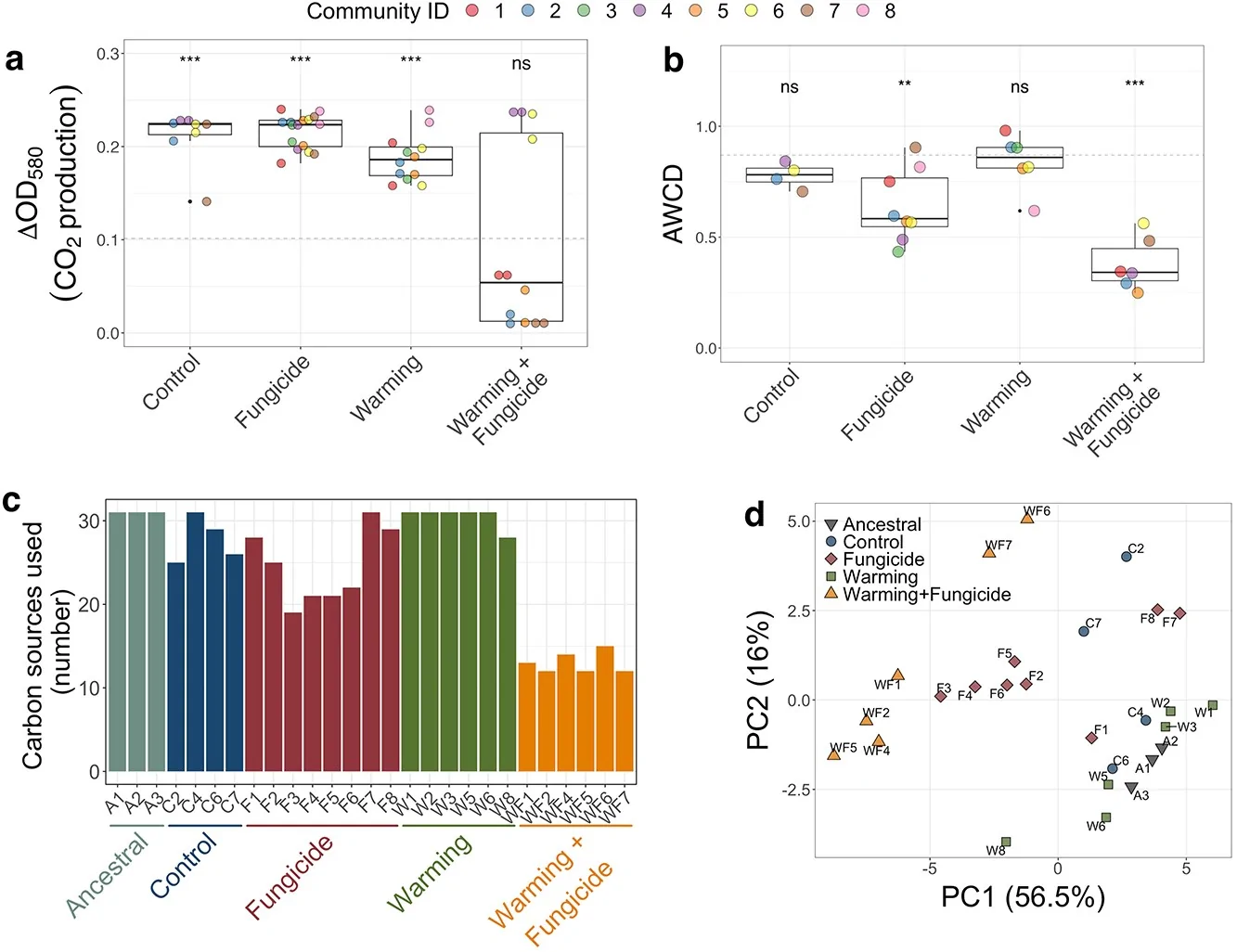
(a–d) Measurements of community functioning for Control, Fungicide-only, Warming-only and Warming+Fungicide treated week-16 communities (a) Community respiration measured as CO₂ production. (b) AWCD determined from Biolog EcoPlate assays. The median (central line), interquartile range (box), and 1.5 × interquartile range (whiskers) are shown. Individual points represent individual communities and are colour-coded by community ID. Asterisks indicate comparisons with the ancestral community (ns = not significant). Significance levels are denoted as: * *P* < .05, ** *P* < .01, *** *P* < .001, **** *P* < .0001. Grey dashed horizontal line shows mean ancestral value (c) Number of metabolized carbon sources (maximum = 31) for ancestral and week-16 communities. X-axis labels show a single letter C, F, W, or WF (corresponding to Control, Fungicide-only, Warming-only or Warming+Fungicide, respectively), followed by community ID (1–8). (d) PCoA of community-level substrate utilization profiles.

We measured CO_2_ production as a proxy for community productivity for week-16 and ancestral communities. Productivity was best explained by a significant fungicide × warming interaction (GLS: *×*^2^*_1_* = 6.11, *P* < .05; Fig. 2a), with combined Warming+Fungicide communities showing lower CO_2_ production than expected from individual effects of warming and fungicide (i.e. a non-additive, synergistic interaction). Warming+Fungicide communities had reduced CO_2_ production relative to Fungicide-only communities (Dunn test, *P.* adj < .05), but no significant difference in CO_2_ production was found between Warming+Fungicide versus Control or Warming+Fungicide versus Warming-only communities (Dunn test, *P.* adj > .05). However, there was notable variation in CO_2_ production among Warming+Fungicide communities, with two communities (WF4 and WF6) retaining high CO_2_ production, and the remaining Warming+Fungicide communities having very low CO_2_ production. This heterogeneity likely contributed to the lack of significant differences in some pairwise comparisons. The mean change in CO_2_ production relative to Controls was +2.37% for Fungicide-only, −10.9% for warming-only, and −64.5% for Warming+Fungicide communities.

Community CO_2_ production under Control, Fungicide-only and Warming-only treatments significantly exceeded that of the ancestral community (Wilcoxon test, *P.* adj < .001) whereas CO_2_ production for Warming+Fungicide communities did not differ from the ancestral community (Wilcoxon test, *P.* adj > .05). Hence*,* although communities typically increased productivity following 16-weeks of experimental evolution, this increase was not observed for Warming+Fungicide communities.

### Treatment-specific differences in metabolic capacity

AWCD values among our week-16 communities depended on an interaction between fungicide and warming, where the combined Warming+Fungicide communities had lower AWCD values than expected from Fungicide-only or Warming-only values (LM: Fungicide x Warming, *F*_1,20_ = 5.01, *P* < .05, Fig. 2b) (i.e. a non-additive, synergistic interaction). Warming+Fungicide communities had reduced AWCD values compared to Control (TukeyHSD, *P* < .001), Fungicide-only (TukeyHSD, *P* < .01) and Warming-only (Tukey HSD, *P* < .001) communities, whereas no difference was observed between Control and Warming-only or Fungicide-only treatments (TukeyHSD, *P* > .05 in both cases).

Fungicide-only and Warming+Fungicide communities had significantly lower AWCD relative to the ancestral community (t-test, Fungicide only versus ancestor *P.* adj < .01, Warming+Fungicide versus ancestor *P.* adj < .001). Control communities also had reduced AWCD values relative to the ancestor, although the effect was much weaker (*P.* adj = .05). In contrast, AWCD values did not differ between Warming-only communities and the ancestral community (*P.* adj > .05).

The ancestral community was able to successfully metabolize all 31 substrates. However, there was some loss of metabolism across all treatments in our week-16 communities. Control and Warming-only communities had the largest number of metabolized substrates, ranging from 25–31 and 28–31, respectively (Fig. 2c). in contrast, Fungicide-only communities had a mean substrate utilization number of 25, whereas Warming+Fungicide treated communities had a mean substrate utilization number of 13 (Fig. 2c). This pattern was potentially driven by loss of carbohydrate and carboxylic acid metabolism in the fungicide treated communities, especially in Warming+Fungicide treated communities (Table  S3).

Ancestral, Control, Fungicide-only, Warming-only, and Warming+Fungicide communities differed significantly in terms of community-level substrate use (PERMANOVA, *F*_4,22_ = 9.2, R^2^ = 0.63, *P* = .001). Warming+Fungicide communities had different patterns of substrate use compared to Fungicide-only, Warming-only, Control, and Ancestral communities (Pairwise PERMANOVA, *P*. adj < .05 in each case), with the largest differences in substrate utilization patterns occurring between Warming+Fungicide and Ancestral communities (Pairwise PERMANOVA*, R*  ^2^ = 0.78, *P*. adj = .05). In contrast, neither Fungicide-only, Warming-only nor Ancestral communities had significantly altered substrate profiles compared to Control communities (Pairwise PERMANOVA, *P*. adj > .05 in each case). PCA revealed that Warming+Fungicide communities grouped along PC1, which explained 56.7% of the variation in substrate use, although communities WF6 and WF7, were separated from the main group (Fig. 2b). The substrates driving the largest treatment-specific differences were N-Acetyl-D-Glucosamine OD (ANOVA; *F*_4_ = 11.8, *P.* adj < .001), D,L-a-Glycerol Phosphate (ANOVA; *F*_4_ = 14.8, *P.* adj < .001), D-Glucosaminic Acid OD (ANOVA; *F*_4_ = 49.6, *P.* adj < .001), Glycyl-L-Glutamic Acid (ANOVA; *F*_4_ = 9.1, *P*. adj <.001), i-Erythritol (ANOVA; *F*_4_ = 11.7, *P.* adj < .001), and a-Ketobutyric Acid (ANOVA; *F*_4_ = 9.04, *P.* adj < .001) with 20/31 substrates showing significant treatment-specific effects (Fig. S3, Table  S4). Differences were mainly due to reduced metabolic activity in combined Warming+Fungicide communities versus Ancestral, Warming-only and Control communities (Fig. S4). Overall, combined Warming+Fungicide communities were clearly associated with a loss of metabolic activity, particularly for carbohydrates and carboxylic acids, that would not have been predicted by looking at the effects of fungicide or warming in isolation.

### Treatment-specific effects on plant growth promotion and aphid suppression

**Figure 3:**
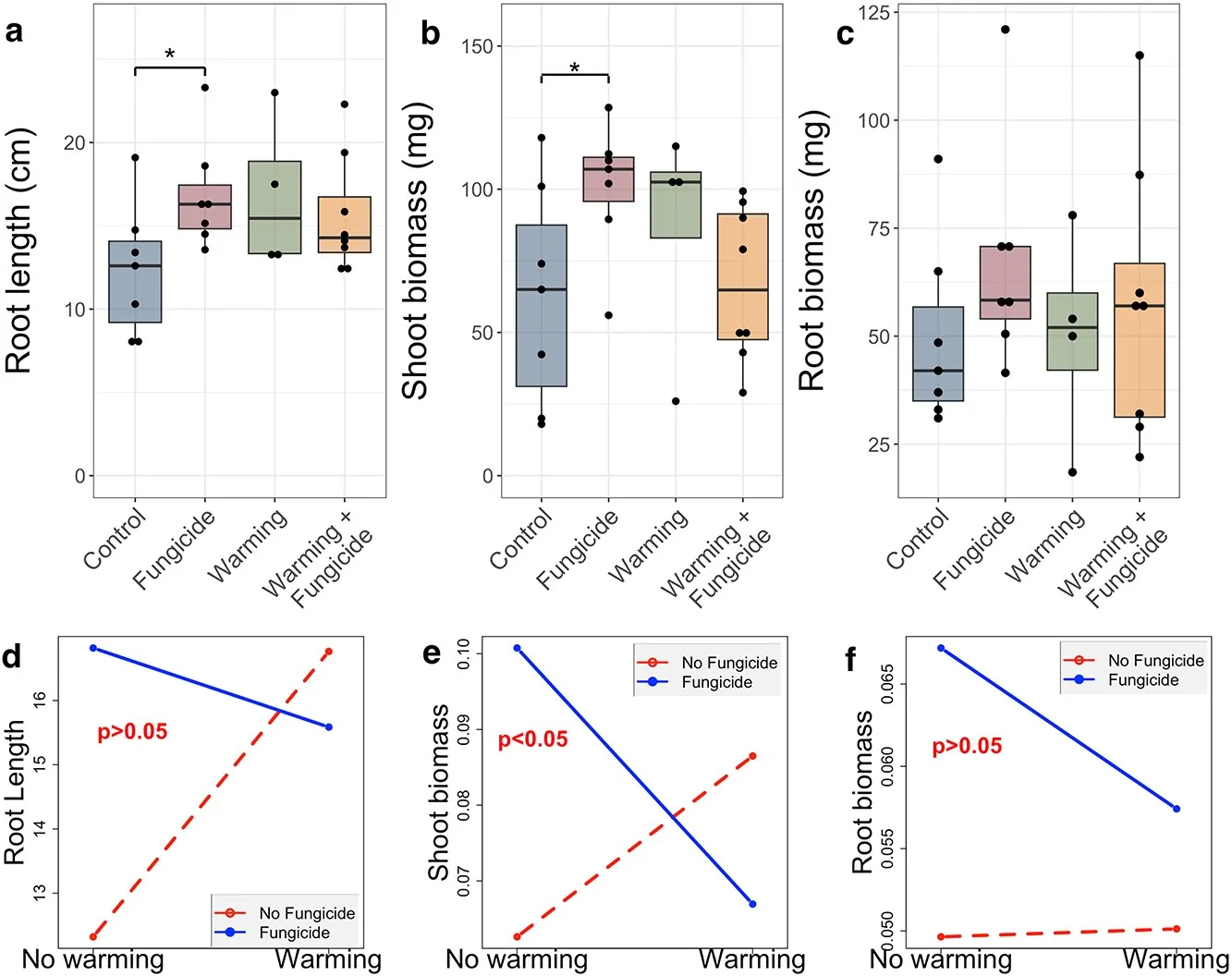
Barley (*Hordeum vulgare*) was grown in sterile compost inoculated with week-16 soil communities, with a history of exposure to Control, Fungicide-only, Warming-only or Warming+Fungicide conditions. Plants were cultivated under common garden control conditions (i.e. 26°C and no added fungicide). Vegetative growth responses are shown at Day 20: (a) root length (b) shoot biomass and (c) root biomass. Each black data point represents the mean of 1–3 replicate plants for an individual community. The median (central line), interquartile range (box), and 1.5 × interquartile range (whiskers) are shown. Between-treatment significance levels (emmeans) are denoted as: * *P* < .05, ** *P* < .01, *** *P* < .001, **** *P* < .0001. Panel d-f shows a single mean value for (d) root length, (e) shoot biomass and (f) root biomass across four soil community treatments. *P* values in red show whether there is a significant fungicide × warming interaction for the corresponding response variable.

We tested whether week-16 soil communities differed in their ability to promote growth during the vegetative stage and aphid defence in barley. Seed germination success did not differ between treatments, with no significant effects of fungicide, warming, or their interaction detected (Binomial GLM: all likelihood ratio tests, *P* > .05). Among plants that germinated, at Day 20, plant performance (root length, shoot biomass, root biomass) was overall shaped by a fungicide x warming interaction (MANOVA fungicide x warming interaction, Pillai’s trace = 0.4, *F*_3,21_ = 4.1, *P* < .05, Fig. 3). Univariate follow-up analyses showed that this interaction was driven primarily by effects on shoot biomass (*F*_1,23_ = 4.9, *P* < .05, Fig. 3b and 3e), with a weaker, marginal effect on root length (*F*_1,23_ = 4.01, *P* = .05, Fig. 3a and d). Pairwise comparisons revealed that Fungicide-only communities conferred increased root length and shoot biomass compared to Control communities (emmeans: *P* < .05, Fig. 3a,b). In contrast, no increase in root length or shoot biomass was observed for Warming+Fungicide communities compared to Control communities (emmeans: *P* > .05).

The effect of fungicide on aphid numbers depended on day (Fungicide x Day, *LRT*_1_ = 7.7, *P* < .01). At Day 16, fungicide treatment was associated with a marginal, but non-significant reduction in aphid numbers (emmeans: *P* = .06, Fig. S5), but this effect became even weaker by Day 20 (emmeans: *P* = .2, Fig. S5). There was no evidence that the fungicide x day interaction depended on warming (NS Fungicide x day x warming, *LRT*_1_ = 1.9, *P* > .05, Fig. S5). Warming alone did not affect aphid numbers (emmeans: *P* > .05, Fig. S5).

### Effect of treatment on fungicide resistance

**Figure 4:**
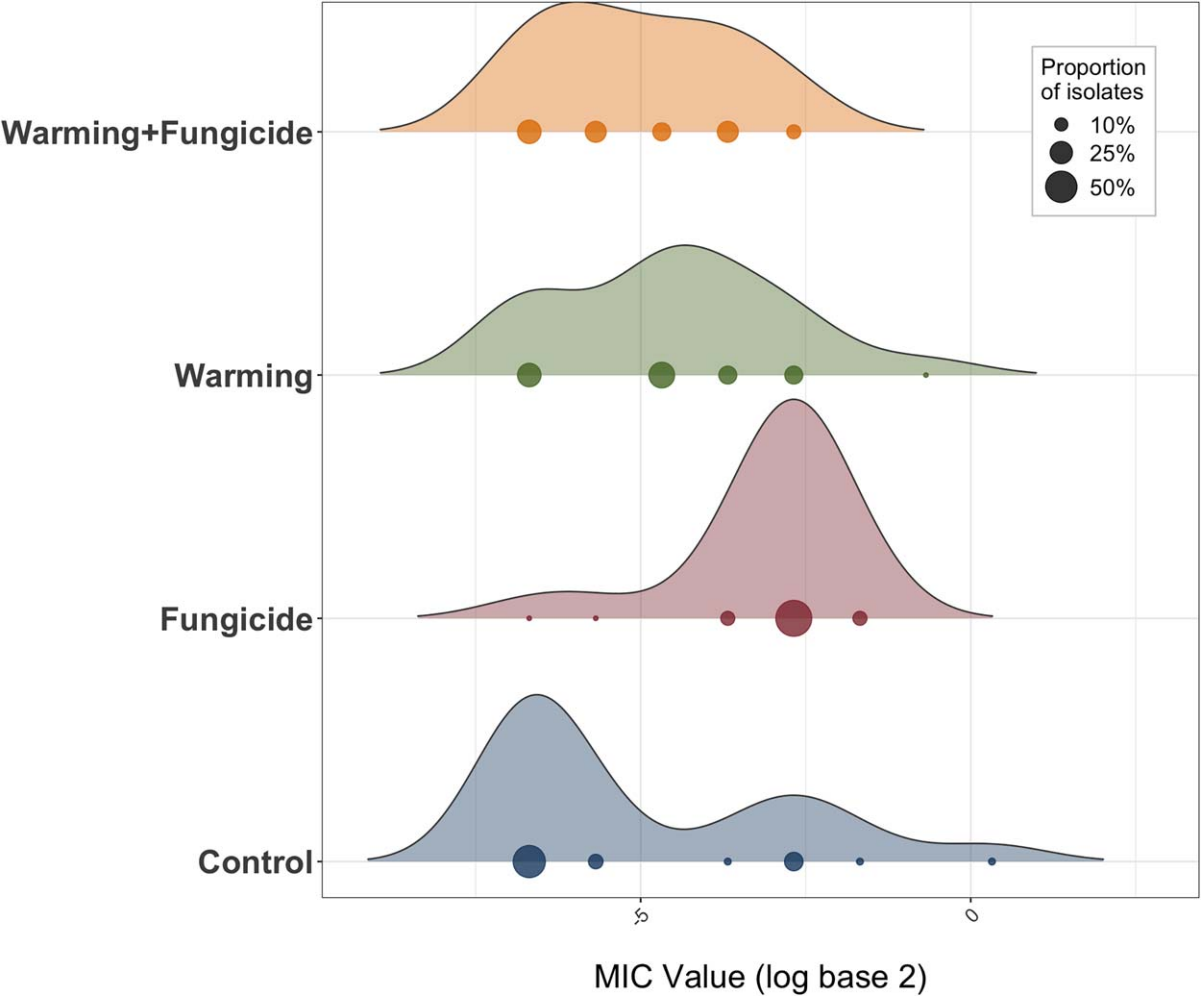
Fungicide resistance (MIC) values for soil isolates recovered from a subsample of week-16 communities. Six isolates were recovered from each of three communities per treatment (Control, Fungicide-only, Warming-only or Warming+Fungicide) and screened for growth in media containing two-fold serial dilutions of Fubol Gold fungicide ranging from 5 mg ml^−1^ to 0.00975 mg ml^−1^. The x-axis shows log₂-transformed mode MIC values. Circle size is proportional to the percentage of isolates displaying a given MIC value.

Fungicide MIC was best explained by an interaction between fungicide and warming, so that soil isolates recovered from fungicide-treated soil were more resistant to fungicide, but this was only true for communities that did not experience warming (LMER; fungicide x warming interaction, *χ*^2^_1,5_ = 5.04, *P* < .05, Fig. 4). For one of our randomly selected communities (W7), our 16 s sequencing could not detect a community, so we re-ran our analyses with W7 omitted and found the same interaction (LMER; fungicide x warming interaction, W7 omitted, *χ*^2^_1,5_ = 4.2, *P* < .05). The mode MIC for isolates from week-16 Fungicide-only communities was 16-fold higher relative to isolates from Control communities (0.0097 mg ml^−1^ versus 0.156 mg ml^−1^), suggesting fungicide exposure increases fungicide resistance in soil communities. Soil isolates from Warming-only communities also increased mode MIC 4-fold relative to isolates from Control communities. However, despite both stressors independently selecting for resistance, the mode MIC of isolates from Warming+Fungicide communities did not differ from Control community isolates (mode MIC = 0.0097 mg ml^−1^ for both treatments). Hence, fungicide selected for soil isolates that were significantly more resistant to the fungicide, but selection for resistance was constrained in the Warming+Fungicide environment.

## Discussion

We explored how two environmental stressors (fungicide and warming temperatures) interact to shape soil bacterial community diversity, composition, adaptation, and functioning. Fungicide was the dominant driver of bacterial community composition, shifting communities toward Gram-negative taxa such as *Burkholderiales, Xanthomonadales, Pseudomonadales*, and *Enterobacterales*, and reducing overall diversity. Functioning assays (CO_2_ production) and assessments of metabolic capacity revealed clear non-additive synergistic effects of fungicide and warming on functioning, so that functioning was greatly decreased in Warming+Fungicide treated communities compared to Warming-only or Fungicide-only communities. Finally, by cultivating *H. vulgare* under common-garden conditions (i.e. under ambient temperature and in the absence of fungicide), we tested whether legacy effects of soil community treatment translated into plant growth metrics. We found weak but significant evidence for a non-additive effect of fungicide and warming on shoot biomass, so that fungicide-treated soil communities increased shoot biomass, but only when soil communities had no history of warming. Together, our results suggest that combined environmental stressors can impair functional capacity beyond the sum of individual effects.

In our study, fungicide accumulation and warming temperatures synergistically reduced community productivity (CO₂ production) and metabolic capacity across 31 carbon sources. Stress adaptation in microbial communities may be broadly shaped by both ecological (i.e. species sorting) and evolutionary processes, and we do not attempt to distinguish here between these two mechanisms operating on our communities. Given the short generation times of bacteria, ecological and microevolutionary responses can occur simultaneously under environmental stress, previously demonstrated in microbial communities exposed to heavy metal contamination [51]. At the ecological scale, synergistic loss of function may reflect each stressor selecting for a different subset of taxa, such that their combination creates sub-optimal conditions for surviving communities. We found that bacterial community composition was distinct for Fungicide-only versus Warming-only communities, suggesting that these two stressors each selected for a distinct subset of taxa, that may have been suboptimal under Warming+Fungicide conditions. This may have also been reflected in our finding that isolates from Warming+Fungicide communities did not display increased resistance to fungicide, unlike isolates form Warming-only communities. However, evolutionary trade-offs could also explain loss of functioning and reduced fungicide resistance in Warming+Fungicide treated communities, if increased adaptation to fungicide comes at the cost of increased susceptibility to warming (i.e. trade-offs [7, 9]). Such constraints on adaptation in multistressor environments are common in evolution experiments [11, 52, 53].

It is also possible that the observed reduction in functioning in Warming+Fungicide communities is partly driven by reduced total microbial abundance. Although we did not directly quantify total community abundance, we included CFU g^−1^ (culturable bacteria) as a covariate in our functional models, and the interaction between fungicide and warming remained significant. Warming+Fungicide communities had ~10-fold lower abundances than Warming-only or Fungicide-only communities, yet abundances remained overall high across all communities (~ 10^8−^10^9^ CFU g^−1^). However, we acknowledge that CFU-based estimates are limited to the culturable fraction of the community, and therefore provide only a coarse proxy for total abundance. Nonetheless, in natural ecosystems, reduced microbial abundance is a biologically meaningful outcome of stress exposure and represents a genuine pathway by which environmental stressors can influence ecosystem functioning.

We found that bacterial communities had reduced α-diversity (i.e. richness and evenness) and distinct bacterial composition following 16 weeks of exposure to fungicide. Our findings are consistent with recent large-scale evidence that pesticide residues are associated with reduced soil biodiversity and altered microbial community composition across European agricultural soils [18]. However, the effects of pesticides on soil bacterial richness are not always consistent [18] and may depend on pesticide class, exposure duration, and indirect effects mediated through other members of the soil microbiome. Fungicides may alter bacterial communities not only through direct toxicity, but also by disrupting fungal populations and modifying competitive and facilitative interactions between fungi and bacteria, potentially leading to cascading shifts in bacterial community structure [54, 55]. Consequently, the reductions in bacterial diversity observed in our study may reflect both direct effects of fungicide exposure and indirect effects arising from alterations to the fungal community. A similar study [56] exposed soil microcosms to environmental disturbance (high heat or humidity) following by pesticide application, reporting strong effects of disturbance on community structure and diversity, with comparably weak effects of pesticide. However in [56] stressors were imposed sequentially, rather than simultaneously.

The composition of the barley root microbiome, which is recruited by plants from bulk soil, is known to influence plant growth and aphid suppression [57]. We found that plants grown in soils with a history of fungicide (Fungicide-only soil communities) had a weak, but significant increase in shoot biomass during the early vegetative stage, which is known to correlate with later plant health [39]. In contrast, when seeds were planted in Warming+Fungicide treated soil communities, this growth promotion effect of fungicide was lost. One possible explanation is that fungicide reduced the prevalence of plant fungal pathogens, maintaining community functioning in the absence of warming (although we did not characterize the fungal community in this study). However, in Warming+Fungicide treated soils, the benefits of reduced fungal pathogen load may be offset by massive losses in community functioning. Our plants were cultivated under common garden conditions (in absence of fungicide or warming), suggesting these stress legacy effects can influence aboveground processes. Such legacy effects of stress have been reported previously for soil microbial communities [2], where ecological memory of soil communities can affect ecosystem resilience. Despite previous research showing plant aphid defences depend on belowground beneficial soil bacteria [58], we did not see any clear treatment-specific effects on aphid numbers, at least during the early vegetative plant growth stage tested in our experiment. Finally, seed germination was relatively low (~50%) across all treatments. Although germination rate did not differ between treatment groups, this suggests that the common-garden plant growth conditions (26°C) may have been slightly above the optimal temperature range for our barley cultivar (15°C–20°C [44]). We chose to run the plant growth experiment at 26°C because this was the control temperature used during our 16-week evolution experiment. Because all plants were grown under identical common garden conditions, this limitation does not affect comparisons among soil community treatments. However, we acknowledge that plant responses to temperature can be mediated through interactions with the soil microbiome [59], and future studies could explore how warming-induced changes in microbial communities influence plant performance across a broader range of growth temperatures.

Although the individual effects of fungicide and warming on soil communities have been examined across a range of experimental systems, soil types, warming regimes, and pesticide classes [17, 18, 20–22, 60, 61], evidence increasingly suggests that the number of stressors has a greater influence on soil ecosystem functioning than the identity of individual stressors [2, 5]. Experimental constraints associated with multistressor designs remain a major challenge. Hierarchical approaches that prioritize stressors with the largest predicted impacts, combined with random partition designs (e.g. [62]) may help disentangle interactive effects. Integrating laboratory microcosms with field-based manipulations across diverse soil types will further improve ecological realism and help guide management strategies to maintain soil health under global change.

## Supplementary Material

Supplementary_material_wrag196

## Data Availability

Sequencing data is available at NCBI (ID PRJNA1475048). All data associated with this manuscript are available in the supplementary material.
