# Supplementary material for: Fungicide and warming interact to reduce soil ecosystem functioning
Source: ISME J. 2026 Jul 26;20(1):wrag196. doi: 10.1093/ismejo/wrag196 (PMC13552498; doi:10.1093/ismejo/wrag196)
Supplement: Supplementary file 1 [file supplementary_material_wrag196.zip › Supplementary figures and tables R3_wrag196.pdf]

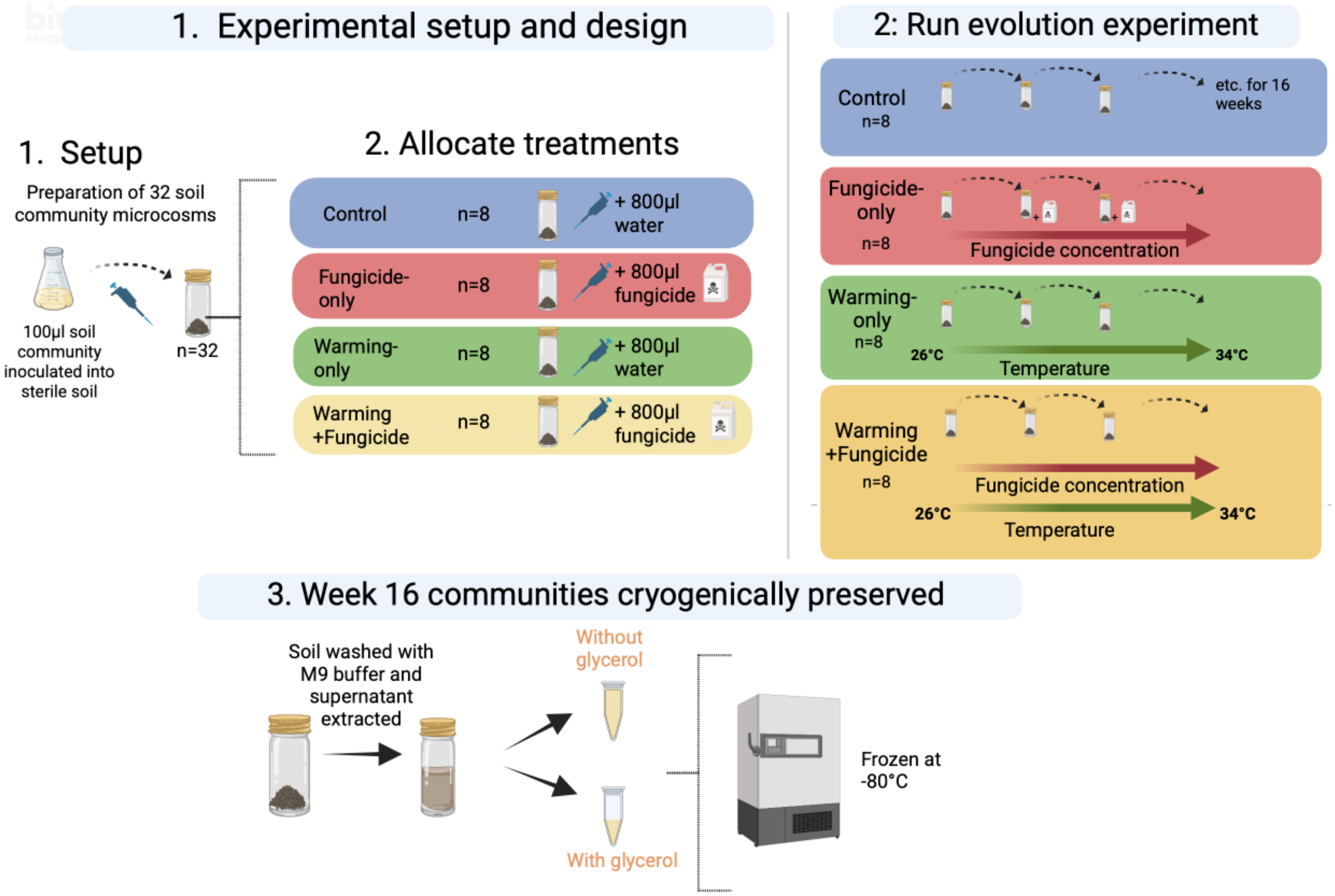
1. Experimental setup and design
1. Setup
Preparation of 32 soil community microcosms
100µl soil community inoculated into sterile soil
n=32
2. Allocate treatments
Control
n=8
+ 800µl water
Fungicide-only
n=8
+ 800µl fungicide
Warming-only
n=8
+ 800µl water
Warming +Fungicide
n=8
+ 800µl fungicide
2: Run evolution experiment
Control
n=8
etc. for 16 weeks
Fungicide-only
n=8
Fungicide concentration
Warming-only
n=8
26°C
34°C
Temperature
Warming +Fungicide
n=8
Fungicide concentration
26°C
34°C
Temperature
3. Week 16 communities cryogenically preserved
Soil washed with M9 buffer and supernatant extracted
Without glycerol
With glycerol
Frozen at -80°C

Supplementary Figure S1: Experimental design for 16-week evolution experiment (Section 2.1). **1.** Thirty-two 30g glass universals each containing 10 g sterilised John Innes No.2 compost were inoculated with 100 μl community soil supernatant ("Ancestral community"). The 32 microcosm communities were then divided into 4 treatment groups: Control, Fungicide-only, Warming-only and Fungicide+Warming, giving 8 replicate communities per treatment. Fungicide solution was added to communities from Fungicide-only (n=8) and Fungicide+Warming (n=8) treatments, and the same volume of water was added to Control (n=8) and Warming-only (n=8) treatments. **2.** Communities were passaged for 16-weeks, passaging to fresh soil (spiked with fresh fungicide solution or water, where appropriate) every 7 days. For fungicide-treated communities (Fungicide-only and Fungicide+Warming treatments), fungicide concentration increased every 4 weeks. All communities were incubated at 26°C at day 0, however for warming-treated communities (Warming-only and Fungicide+Warming treatments), temperature increased by 0.5°C every week. **3.** At the end of the experiment (week 16), all 32 soil community supernatants were cryogenically preserved at -80°C. Created in BioRender. Obrien, S. (2026) (https://BioRender.com/xoa1ajz).

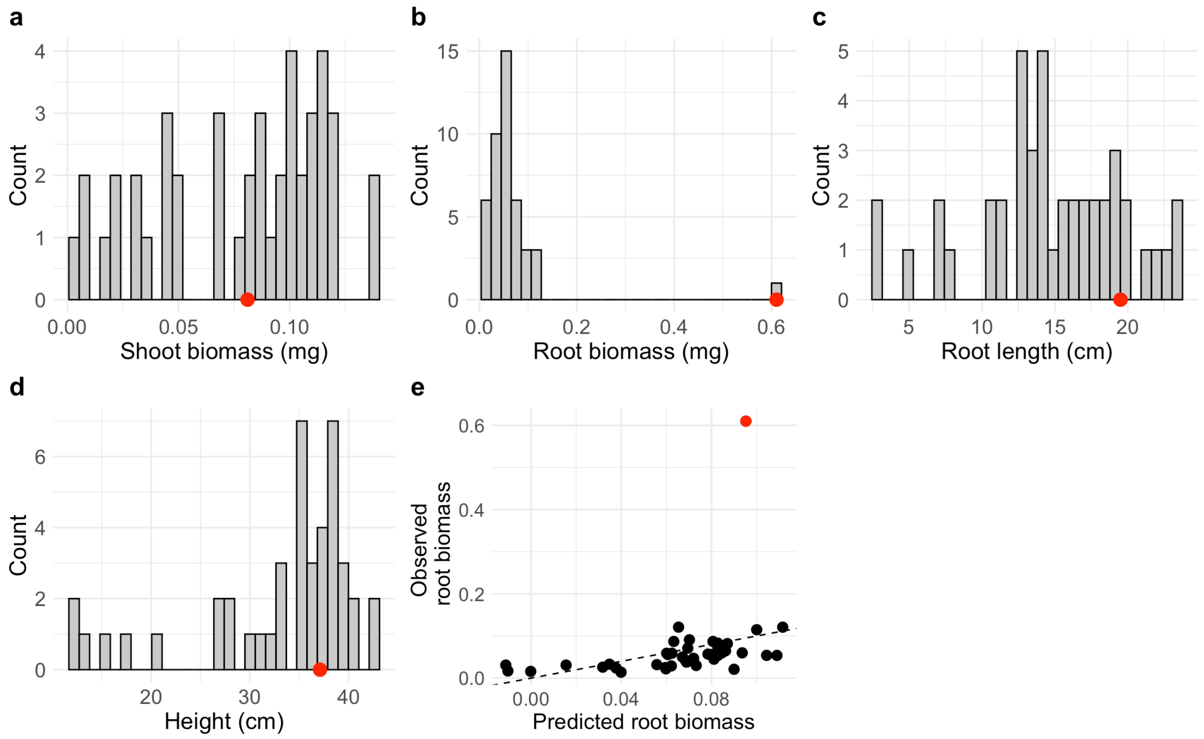

Supplementary Figure S2: To confirm two root biomass values (ID 29 and 62; **panel b,** shown as red points) likely reflected measurement error and were therefore true outliers, we examined the height, shoot biomass, and root length values for these two plants, which all fell within the normal range of the full dataset (**panels a, c and d;** plants 29 and 62 shown as red points). We also fit a linear model predicting root biomass from other plant traits (i.e. Root biomass ~ Height + Shoot biomass + Root length). For plants 29 and 62, observed root biomass values were substantially higher than predicted values (**panel e,** values for plants 29 and 62 shown in red). Plants 29 and 62 were therefore removed from the dataset.

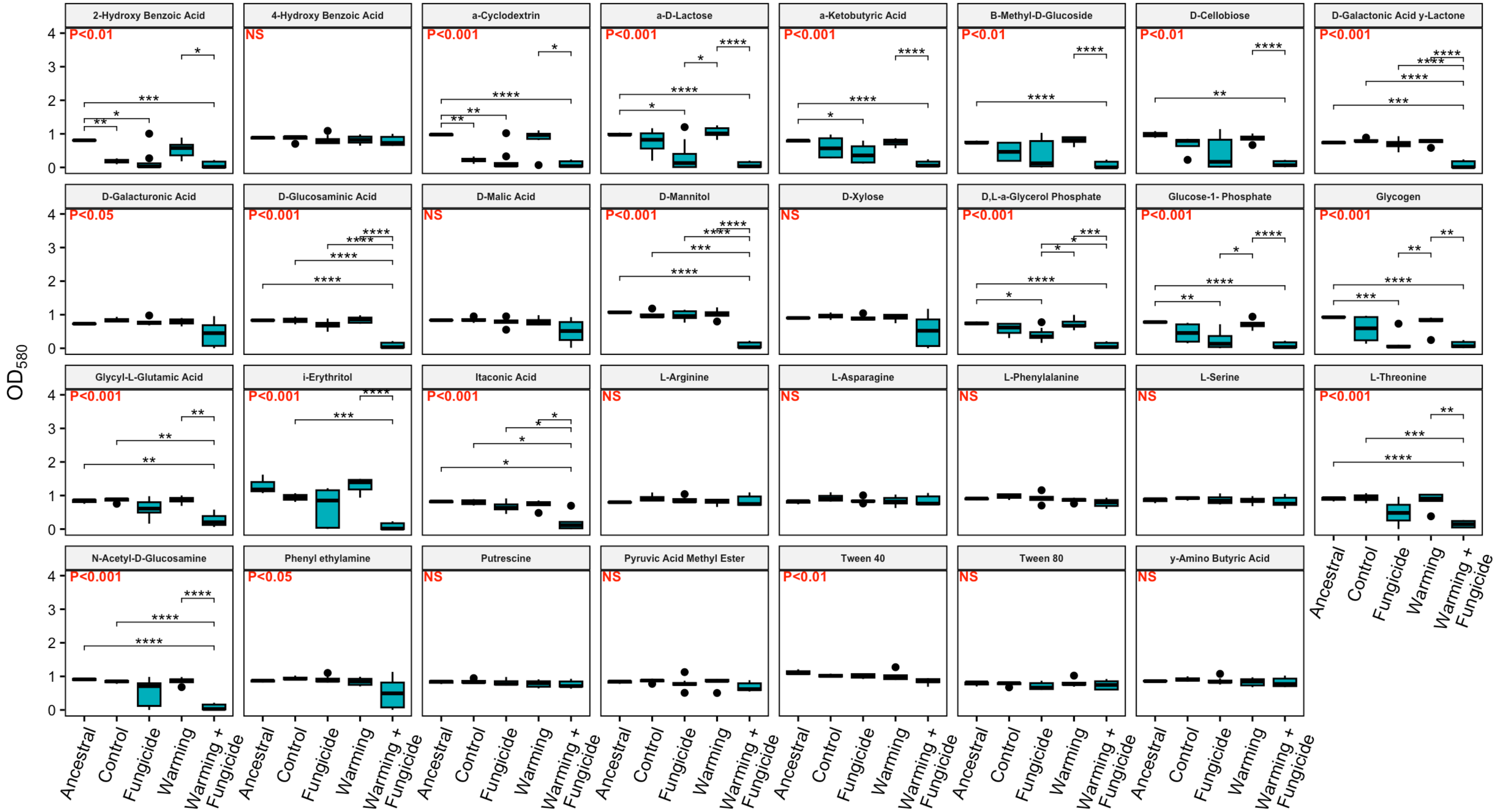

Supplementary Figure S3. **F**or each Biolog carbon source ,we quantified treatment-specific differences in metabolic activity (OD_{580}) using ANOVA with Benjamini-Hochberg correction and t-tests. ANOVA results (adjusted p-values) are shown in red. Significant t-tests comparisons are shown as black connecting lines. Significance levels are denoted as follows: *P.* adj < 0.05 (*), *P.* adj < 0.01 (**), *P.* adj < 0.001 (***), *P.* adj < 0.0001 (****). Boxplots show the median (central line), interquartile range (box), and 1.5× interquartile range (whiskers).

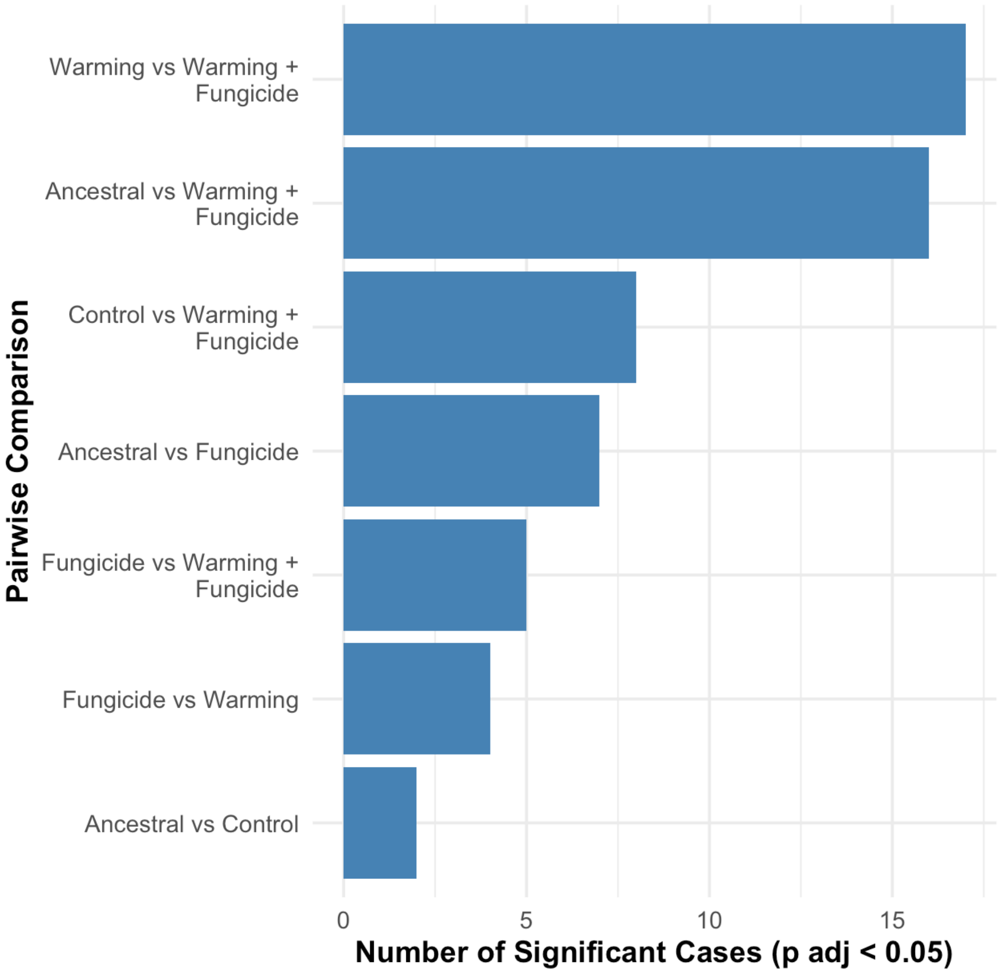

Supplementary Figure S4. Treatment-specific differences in metabolic activity were found for 20 out of 31 Biolog carbon sources. To identify the dominant treatment-level comparison driving these effects, we show the number of significant cases for each comparison across all 31 carbon sources. The dominant comparisons driving treatment-specific effects were reduced activity in warming + fungicide communities versus warming-only communities (effect observed for 17/31 carbon sources), warming + fungicide communities versus ancestral communities (effect observed for 16/31 carbon sources), and warming + fungicide communities versus control communities (effect observed for 8/31 carbon sources) communities.

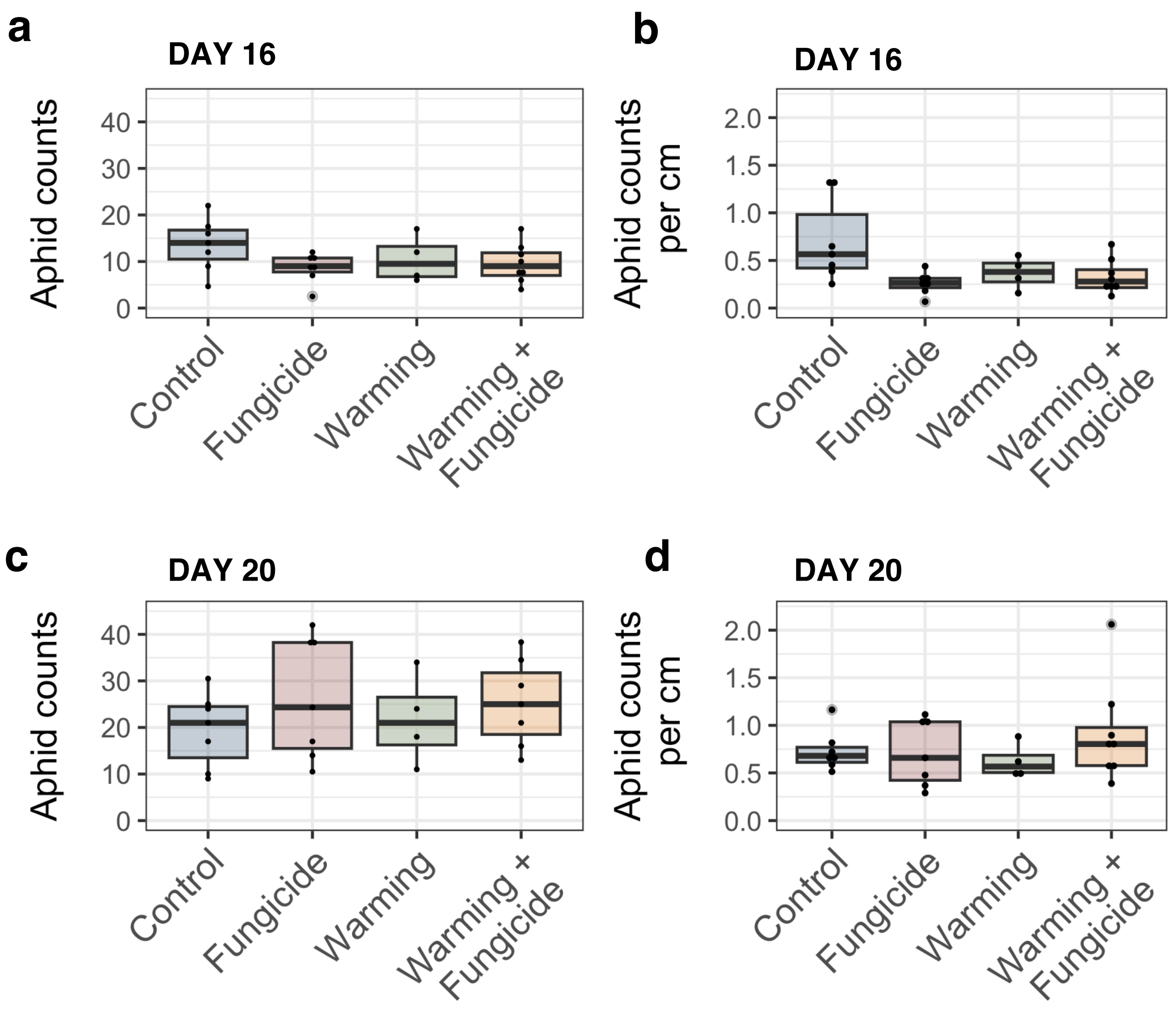

Supplementary Figure S5: Aphid abundance (numbers) on barley plants grown compost inoculated with week-16 microbial communities. Barley seeds were inoculated at planting, and aphids were introduced on day 6. Adult aphids were counted on day 16 (**panels a & b**) and day 20 (**panels c & d**). Aphid data is presented as counts (**panels a & b**) and as counts divided by cm plant height (**panels c & d**). Each datapoint represents rounded mean aphid number per soil community (1–3 replicates per community). Boxplots show the median (central line), interquartile range (box), and 1.5× interquartile range (whiskers).

Supplementary Table S1: Thirty-one Carbon sources tested using Biolog EcoPlates. Control wells contain water.

Well	Carbon Source	Category
A1	Water (Control)	Control
A2	β-Methyl-D-Glucoside	Carbohydrate
A3	D-Galactonic Acid γ-Lactone	Carbohydrate
A4	L-Arginine	Amino Acid
A5	Pyruvic Acid Methyl Ester	Carboxylic Acid
A6	D-Xylose	Carbohydrate
A7	D-Galacturonic Acid	Carboxylic Acid
A8	L-Asparagine	Amino Acid
A9	Tween 40	Polymer
B1	Tween 80	Polymer
B2	α-Cyclodextrin	Polymer
B3	Glycogen	Polymer
B4	D-Cellobiose	Carbohydrate
B5	α-D-Lactose	Carbohydrate
B6	β-Methyl-D-Glucoside	Carbohydrate
B7	D-Mannitol	Carbohydrate (Sugar Alcohol)
B8	N-Acetyl-D-Glucosamine	Carbohydrate (Amino Sugar)
B9	D-Glucosaminic Acid	Carboxylic Acid
C1	D-Glucose-1-Phosphate	Carbohydrate
C2	D-Glucose-6-Phosphate	Carbohydrate
C3	D-Galactose	Carbohydrate
C4	D-Fructose	Carbohydrate
C5	D-Mannose	Carbohydrate
C6	Inosine	Nucleoside
C7	Phenylethylamine	Amine
C8	Putrescine	Amine
C9	2-Hydroxybenzoic Acid (Salicylic Acid)	Phenolic Compound
D1	4-Hydroxybenzoic Acid	Phenolic Compound
D2	γ-Hydroxybutyric Acid	Carboxylic Acid
D3	Itaconic Acid	Carboxylic Acid
D4	α-Ketobutyric Acid	Carboxylic Acid
D5	DL-α-Glycerol Phosphate	Carbohydrate Derivative
D6	Glycyl-L-Glutamic Acid	Amino Acid (Dipeptide)
D7	L-Serine	Amino Acid
D8	Phenylalanine	Amino Acid
D9	Control (No Carbon)	Control

Supplementary Table S2: The relative abundances (green bars) of taxa (Family) in the table below differed significantly between treatments (Control, Fungicide-only, Warming-only and Warming+Fungicide). W = ANCOM statistic indicating the number of times the null hypothesis of no treatment-specific differences was rejected.

Taxa	Control	Warming-only	Fungicide-only	Warming+ Fungicide	*W*
Pseudomonadaceae	6.89	2.6	23.2	15.6	152
Enterobacteriaceae	3.2	0.8	13.1	9.8	157
Alcaligenaceae	5.2	4.6	8.6	12.5	138
Rhodanobacteraceae	1.2	0.5	5	11.3	153
Rhizobiaceae	2	1.8	4.1	6.4	153
Microbacteriaceae	1.3	1.5	2.6	6.9	144
Sphingomonadaceae	0.7	0.7	1.5	0	157
Paenibacillaceae	16.4	13.6	0	1.1	151
Chitinophagaceae	3.4	2.8	0	0.7	138
Nocardioidaceae	3.2	1.9	0	0.2	145
Bacillaceae	7.8	19.7	0	0	157
Streptomycetaceae	1.6	9.9	0	0	153
Comamonadaceae	1.5	0.8	0	0	152
Brevibacillaceae	1.5	2.3	0	0	151
Isosphaeraceae	2.1	3	0	0	150
Flavobacteriaceae	2.2	0.3	0	0	131
Devosiaceae	1.3	1.3	0	0	129
Other	61.49	68.1	58.1	64.5	

Supplementary Table S3. To assess how fungicide and warming affected community metabolic capacity, we evaluated the ability of evolved microbial communities to metabolize 31 different carbon sources over an 8-day incubation period. The table presents data from day 8, showing the proportion of communities within each treatment group (Control, Fungicide-only, Warming-only and Warming+Fungicide) that retained the ability to metabolize each carbon source (defined as Ci - R > 0.25).

	Ancestor (n=4)	Control (n=4)	Fungicide (n=8)	Warming (n=6)	Warming + Fungicide (n=6)
**Amines**					
Phenyl ethylamine	1	1.00	1.00	1.00	0.50
Putrescine	1	1.00	1.00	1.00	1.00
**Amino acids**					
Glycyl-L-Glutamic Acid	1	1.00	0.88	1.00	0.50
L-Arginine	1	1.00	1.00	1.00	1.00
L-Asparagine	1	1.00	1.00	1.00	1.00
L-Phenylalanine	1	1.00	1.00	1.00	1.00
L-Serine	1	1.00	1.00	1.00	1.00
L-Threonine	1	1.00	0.75	1.00	0.17
**Carbohydrates**					
a-D-Lactose	1	0.75	0.38	1.00	0.00
B-Methyl-D-Glucoside	1	0.50	0.38	1.00	0.00
D-Cellobiose	1	0.75	0.50	1.00	0.00
D-Galactonic Acid y-Lactone	1	1.00	1.00	1.00	0.00
D-Mannitol	1	1.00	1.00	1.00	0.00
D-Xylose	1	1.00	1.00	1.00	0.50
D,L-a-Glycerol Phosphate	1	1.00	0.75	1.00	0.00
Glucose-1- Phosphate	1	0.50	0.38	1.00	0.00
i-Erythritol	1	1.00	0.62	1.00	0.00
N-Acetyl-D-Glucosamine	1	1.00	0.62	1.00	0.00
**Carboxylic acids**					
a-Ketobutyric Acid	1	1.00	0.62	1.00	0.00
D-Galacturonic Acid	1	1.00	1.00	1.00	0.50
D-Glucosaminic Acid	1	1.00	1.00	1.00	0.00
D-Malic Acid	1	1.00	1.00	1.00	0.67
Itaconic Acid	1	1.00	1.00	1.00	0.17
Pyruvic Acid Methyl Ester	1	1.00	1.00	1.00	1.00
y-Amino Butyric Acid	1	1.00	1.00	1.00	1.00
**Phenolic compounds**					
2-Hydroxy Benzoic Acid	1	0.25	0.25	0.83	0.00
4-Hydroxy Benzoic Acid	1	1.00	1.00	1.00	1.00
**Polymers**					
a-Cyclodextrin	1	0.25	0.25	0.83	0.00
Glycogen	1	0.75	0.12	0.83	0.00
Tween 40	1	1.00	1.00	1.00	1.00
Tween 80	1	1.00	1.00	1.00	1.00

Supplementary Table S4: For each carbon source we quantified loadings on PC1 and tested for treatment-specific differences using ANOVA (F-value))with Benjamini-Hochberg correction (Adj. p-value). Carbon sources are listed in the order of their greatest contributors to treatment-specific differences (highest- lowest).

Carbon Source	F-value	df	p-value	Adj. p-value	PC1	PC2
N-Acetyl-D-Glucosamine	11.78	4	2.9e-05	1.3e-04	0.2176	-0.0865
D,L-a-Glycerol Phosphate	14.77	4	5.3e-06	4.1e-05	0.2169	-0.1375
D-Glucosaminic Acid	49.63	4	1.1e-10	1.6e-09	0.2163	-0.0692
Glycyl-L-Glutamic Acid	9.05	4	1.8e-04	3.9e-04	0.2122	-0.0620
i-Erythritol	11.71	4	3.0e-05	1.3e-04	0.2120	-0.1072
a-Ketobutyric Acid	9.04	4	1.8e-04	3.9e-04	0.2097	-0.1535
D-Galactonic Acid y-Lactone	40.66	4	7.3e-10	7.6e-09	0.2073	-0.0418
B-Methyl-D-Glucoside	5.87	4	2.3e-03	4.1e-03	0.2059	-0.1315
D-Mannitol	64.61	4	7.7e-12	2.4e-10	0.2040	-0.0501
L-Threonine	11.09	4	4.4e-05	1.5e-04	0.1990	-0.1010
Itaconic Acid	12.86	4	1.5e-05	9.4e-05	0.1985	-0.0655
Glucose-1- Phosphate	9.65	4	1.2e-04	3.0e-04	0.1979	-0.1820
D-Cellobiose	7.23	4	7.1e-04	1.5e-03	0.1915	-0.1503
a-D-Lactose	10.06	4	8.7e-05	2.5e-04	0.1865	-0.2127
Pyruvic Acid Methyl Ester	1.23	4	3.3e-01	4.2e-01	0.1856	0.1820
a-Cyclodextrin	10.18	4	8.1e-05	2.5e-04	0.1812	-0.1099
Glycogen	11.44	4	3.6e-05	1.4e-04	0.1801	-0.1700
2-Hydroxy Benzoic Acid	6.89	4	9.4e-04	1.8e-03	0.1749	-0.0876
D-Galacturonic Acid	3.73	4	1.8e-02	3.0e-02	0.1652	0.0757
Tween 40	5.15	4	4.4e-03	7.6e-03	0.1648	-0.0111
L-Phenylalanine	2.57	4	6.6e-02	8.9e-02	0.1611	0.2050
4-Hydroxy Benzoic Acid	0.32	4	8.6e-01	8.6e-01	0.1603	0.2825
Putrescine	0.70	4	6.0e-01	7.1e-01	0.1581	0.2975
L-Serine	0.49	4	7.5e-01	7.7e-01	0.1546	0.2940
Phenyl ethylamine	3.24	4	3.1e-02	4.8e-02	0.1540	0.1044
D-Xylose	3.01	4	4.0e-02	5.7e-02	0.1537	0.0673
y-Amino Butyric Acid	0.52	4	7.2e-01	7.7e-01	0.1452	0.3262
D-Malic Acid	3.02	4	4.0e-02	5.7e-02	0.1429	0.0147
Tween 80	0.68	4	6.2e-01	7.1e-01	0.1211	0.1542
L-Asparagine	0.60	4	6.7e-01	7.4e-01	0.1070	0.3568
L-Arginine	0.76	4	5.6e-01	6.9e-01	0.0899	0.3681

p-values adjusted by Benjamini–Hochberg correction.
