# Supplementary material for: Fungicide and warming interact to reduce soil ecosystem functioning
Source: ISME J. 2026 Jul 26;20(1):wrag196. doi: 10.1093/ismejo/wrag196 (PMC13552498; doi:10.1093/ismejo/wrag196)
Supplement: Supplementary file 1 [file supplementary_material_wrag196.zip › Supplementary methods R2_wrag196.pdf]

Supplementary methods

SM1.1 Community experimental evolution

We added 100 μl (10^5 CFU/ml) of *Pseudomonas fluorescens* tagged with a gentamicin-resistant cassette (SBW25::GentR) to each community microcosm at a 1:1 ratio with the community, as we wanted to simultaneously investigate *de novo* adaptation of a focal species *P. fluorescens,* to fungicide and warming. However, this strain was quickly outcompeted by the community and could not be retrieved from the community by the end of the 16 week experiment.

SM1.2 Preparation of respiration detection lid

The respiration detection lid was prepared as follows. Indicator agar was produced by first preparing an indicator solution containing 16.77 g KCl (Sigma-Aldrich), 18.75 mg Cresol Red (Acros), and 0.315 g $NaHCO_3$ (Sigma) in 1 l of distilled water heated to 50 °C in a water bath. A solution of 3% (w/v) Nobel agar (Sigma-Aldrich) was prepared in distilled water and heated in a microwave until completely dissolved. Agar was left in a water bath to cool to 50 °C, and then indicator solution was added to the agar in a 2:1 ratio (Indicator:agar) and gently stirred until homogenised. 150 μl of agar was added to each well of a 96-well plate (Thermo Scientific) and left to solidify. To standardise the OD of agar in each well, plates were sealed in an anaerobic jar containing soda lime (Thermo Scientific) and a beaker of water. After 48 hours, the plates were sealed with parafilm and returned to the anaerobic jar. The OD (580 nm) of each well was measured at the start and end of the experiment using a Tecan Nano Plate reader.

SM1.3 Colony isolation for fungicide MIC assay

We chose 3 communities at random per treatment for fungicide resistance assays on individual isolates: Control (C4, C5, C7), Fungicide (F1, F2, F5), Warming (W1, W2, W7) and Warming+Fungicide (WF1, WF4, WF5). Frozen community stocks were re-established in 10 g fresh sterile soil for 7 days. A soil wash was extracted in PBS, diluted, and plated on Lysogeny broth agar (LB; Fisher Scientific), Reasoner's2A agar (R2A; Fisher Scientific), and Tryptone Soy Agar (TSA; Fisher Scientific) plates. Plates were incubated at 28°C for 7 days. A variety of agar types were used to isolate a variety of bacteria from each of the evolved communities, rather than selecting for bacteria that are fast growing and only grow on one type of agar. Colonies were selected randomly where possible, with the aim of obtaining representatives from each medium. The table below shows the agar type each isolate was selected from across all 12 communities.

Source	Community ID	Isolate number	Agar type
Control	C4	**1**	Tryptone Soy
Control	C4	**2**	Tryptone Soy
Control	C4	**3**	Tryptone Soy
Control	C4	**4**	R2A
Control	C4	**5**	R2A
Control	C4	**6**	LB
Control	C5	**1**	Tryptone Soy
Control	C5	**2**	Tryptone Soy
Control	C5	**3**	R2A
Control	C5	**4**	LB
Control	C5	**5**	LB
Control	C5	**6**	Tryptone Soy
Control	C7	**1**	Tryptone Soy
Control	C7	**2**	LB
Control	C7	**3**	R2A
Control	C7	**4**	R2A
Control	C7	**5**	Tryptone Soy
Control	C7	**6**	Tryptone Soy
Fungicide	F1	**1**	Tryptone Soy
Fungicide	F1	**2**	Tryptone Soy
Fungicide	F1	**3**	R2A
Fungicide	F1	**4**	R2A
Fungicide	F1	**5**	R2A
Fungicide	F1	**6**	LB
Fungicide	F2	**1**	Tryptone Soy
Fungicide	F2	**2**	LB
Fungicide	F2	**3**	R2A

Fungicide	F2	**4**	Tryptone Soy
Fungicide	F2	**5**	R2A
Fungicide	F2	**6**	LB
Fungicide	F5	**1**	R2A
Fungicide	F5	**2**	R2A
Fungicide	F5	**3**	Tryptone Soy
Fungicide	F5	**4**	Tryptone Soy
Fungicide	F5	**5**	LB
Fungicide	F5	**6**	LB
Warming	W1	**1**	Tryptone Soy
Warming	W1	**2**	Tryptone Soy
Warming	W1	**3**	R2A
Warming	W1	**4**	LB
Warming	W1	**5**	LB
Warming	W1	**6**	Tryptone Soy
Warming	W2	**1**	LB
Warming	W2	**2**	LB
Warming	W2	**3**	Tryptone Soy
Warming	W2	**4**	R2A
Warming	W2	**5**	Tryptone Soy
Warming	W2	**6**	Tryptone Soy
Warming	W7	**1**	R2A
Warming	W7	**2**	R2A
Warming	W7	**3**	Tryptone Soy
Warming	W7	**4**	Tryptone Soy
Warming	W7	**5**	Tryptone Soy
Warming	W7	**6**	Tryptone Soy
Fungicide+Warming	WF1	**1**	LB
Fungicide+Warming	WF1	**2**	Tryptone Soy
Fungicide+Warming	WF1	**3**	R2A
Fungicide+Warming	WF1	**4**	LB
Fungicide+Warming	WF1	**5**	R2A
Fungicide+Warming	WF1	**6**	LB
Fungicide+Warming	WF4	**1**	Tryptone Soy
Fungicide+Warming	WF4	**2**	LB
Fungicide+Warming	WF4	**3**	R2A
Fungicide+Warming	WF4	**4**	Tryptone Soy
Fungicide+Warming	WF4	**5**	Tryptone Soy
Fungicide+Warming	WF4	**6**	Tryptone Soy
Fungicide+Warming	WF5	**1**	Tryptone Soy
Fungicide+Warming	WF5	**2**	Tryptone Soy
Fungicide+Warming	WF5	**3**	Tryptone Soy
Fungicide+Warming	WF5	**4**	Tryptone Soy
Fungicide+Warming	WF5	**5**	LB
Fungicide+Warming	WF5	**6**	LB
